# Differences in Process Management and In-Hospital Delays in Treatment with iv Thrombolysis

**DOI:** 10.1371/journal.pone.0075378

**Published:** 2013-09-12

**Authors:** Julia Ferrari, Michael Knoflach, Leonhard Seyfang, Wilfried Lang

**Affiliations:** 1 Department of Neurology, Hospital Barmherzige Brueder, Vienna, Austria; 2 Department of Neurology, Innsbruck Medical University, Innsbruck, Austria; 3 Department for Clinical Neurosciences and Preventive Medicine, Danube University, Krems, Austria; 4 Gesundheit Österreich GmbH/BIQG, Vienna, Austria; D'or Institute of Research and Education, Brazil

## Abstract

**Objectives:**

Rapid initiation of intravenous thrombolysis improves patient’s outcome in acute stroke. We analyzed inter-center variability and factors that influence the door-to-needle time with a special focus on process measurements in all Austrian stroke units.

**Methods:**

Case level data of patients receiving intravenous thrombolysis in the Austrian Stroke Unit Registry were enriched with information of a structured questionnaire on center specific process measures of all Austrian stroke units. Influence of case and center specific variables was determined by LASSO procedure.

**Results:**

Center specific median door-to-needle time ranged between 30 and 78 minutes. Between April 2004 and November 2012, 6246 of 57991 patients treated in Austrian stroke units with acute ischemic stroke received intravenous thrombolysis. An onset-to-door time >120 minutes, patients with total anterior circulation stroke, recent year of admission, patient transportation with ambulance crew and emergency physician, the use of point of care tests reduced the door-to-needle time, whereas onset-to-door ≤60 minutes, unknown onset-to-door, patients with an NIHSS ≤4 or posterior circulation stroke, initial admission to a general emergency department, a distant radiology department, primary imaging modality other than plain CT and waiting for the lab results were associated with an increase in door-to-needle time. Case level and center specific factors could explain the inter center variability of door-to-needle times in 31 of 34 stroke units in Austria.

**Conclusions:**

In light of our results it seems crucial that every single stroke center documents and critically reviews possibilities of optimizing practice strategies in acute stroke care.

## Introduction

Intravenous recombinant tissue plasminogen activator (rtPA) is the gold standard therapy for acute ischemic stroke. [1]–[3] It can only be applied up to 4.5 hours within the onset of stroke symptoms [3] and a short onset-to-treatment time (OTT) translates into a better functional outcome [4]. The OTT is composed of onset-to-door time (ODT) and door-to-needle time (DNT). The latter is dependent on the process management of each individual hospital and therefore used as a benchmark for the organization of in-hospital acute stroke care. Highly specialized centers have reported to reduce this in-hospital delay to as low as a median of 20 minutes. [5] Still recent evaluations in the US (Get With The Guidelines Stroke national United States Registry, GWTG-Stroke) [6] and in Eastern European Countries [7] (Safe Implementation of Treatments in Stroke – East Registry, SITS-EAST) have shown only 26.6% and 38% of patients are treated with iv thrombolysis with a DNT below 60 minutes as recommended by national guidelines. Both evaluations demonstrated a high variability between different centers in reaching this goal ranging between 0% to 84%. [6], [7] Our own previous analysis of the Austrian Stroke Unit Registry [8] as well as other evaluations have described multiple factors increasing the DNT like older age, female sex, black race, low or very high stroke severity, presence of prior stroke, short ODT and performing angiography or perfusion imaging prior to thrombolysis [6], [7]. Yet all these factors fail to explain the high inter-center variability. [6], [7] As possible cause differences in stroke management within stroke centers and countries have been proposed. [7] In order to further explore factors that influence the DNT with a special focus on process measurements we supplemented the extensive hospital and case level data (like for example age, sex, clinical syndrome or National Institutes of Health Stroke Scale score (NIHSS)) of the Austrian stroke unit registry with information of a structured questionnaire on process measures in all stroke units in Austria.

## Methods

Since 2003, a growing network of Austrian stroke units have been collecting data on standard characteristics and acute management of all patients with stroke admitted to 34 Austrian stroke units. Data collection and ratings were performed by experienced stroke neurologists using standardized variable definitions and scores. To ensure high quality of data, immediate data entry was obligatory. The web-based database includes online plausibility checks and help. In biannual meetings of stroke neurologists, details about scoring procedures and variable assessment are thoroughly discussed.

In April 2004, the registry was enriched by documentation of the ODT and the DNT, including details and timing of in-hospital management. Definitions of variables in the registry are listed in Table 1 and have been described previously [8]–[10].

**Table 1:** Available variables on patient and center characteristics.

Variable	Levels (the first is used as reference level)
**Case-based characteristics**	
Gender	male, female
Age	(70, 80], [0, 60], (60, 70], (80, 110]
Modified Rankin Scale prior to current event	0, 1, 2, 3, 4, 5
Hypertension	no, yes
Diabetes mellitus	no, yes
Previous stroke	no, yes
Previous heart attack	no, yes
Hypercholesteremia	no, yes
Atrial fibrillation	no, yes
Other cardiac disease	no, yes
Peripheral artery disease	no, yes
Current smoker	no, yes
Onset-to-Door Time	61–120, 0–60, >120, unknown, (minutes)
National Institute of Health Stroke Scale	5–8, 0–4, 9–12, 13–16, 17–20, 21–42
Etiology	microangiopathy, macroangiopathy, cardiogenic embolism, other, unknown
Clinical Syndrome	partial anterior circulation stroke (PACS), lacunar stroke (LACS), total anterior circulation stroke (TACS), posterior circulation stroke (POCS), other
Admission Date	unit: (days since Jan. 1. 2011)/365.25
Ambulance crew with emergency doctor	no, yes
Urinary catheter	no, yes
Working hours, 8–16 h Monday to Friday	no, yes
**Hospital -based characteristics**	
Pre-notification	>75%, < = 25%, 26–50%, 51–75%
Admission	directly to stroke unit, to emergency department with neurologist on duty, to general emergency department
Radiology department	close, distant
Routine-imaging	CT, other
Who escorts the patient to the radiology department	porters’ services, the doctor on duty, other
Other investigations	no, yes
Wait for blood test	no, point of care test, full blood count, other approach
Weigh the patient	no, yes
Where do patients receive thrombolysis	stroke-unit, general emergency department, on CT table
Rates of thrombolysis	medium (10,20]%, low ≤10%, high >20% (of all acute ischemic strokes)
Individual dummy-variable for each stroke unit (pseudonymized)	

Except the admission date, which is coded as years from January 1, 2011, all variables were dummy-coded with the first category serving as reference level.

To obtain detailed information about variables that affect the DNT in Austria, we designed a structured questionnaire containing 11 questions about possible causes for prolonged DNT, both in the pre-hospital phase and in-hospital phase: (1) What percentage of patients arrive via the pre-notification-system? (<25%, <50%, >50%, >75%); (2) Where are patients admitted to in hospital? (directly to the stroke unit, straight to the emergency department with the neurologist on duty, to the general emergency department); (3) Is the stroke unit/emergency department with a neurologist on duty located near the radiology department? (yes - in the same building reachable within 2 minutes, no); (4) What is your routine choice of imaging investigation before thrombolysis? (plain head CT, other); (5) Who escorts the patient to the CT/MRI scanner? (porters’ services, the doctor on duty, other); (6) Are there any other investigations performed before thrombolysis? (e.g. ultrasound, …) (yes, specify:…, no); (7) Do you wait for any blood test results to come back for before initiating systemic thrombolysis? (No. If the history does not suggest that the patient is on oral anticoagulation, thrombolysis is immediately started; point of care tests are routinely performed first; we wait for the full blood count to come back first; any other approach (please state)); (8) Do you weigh the patient on a scale? (yes, no);(9) Where do patients receive thrombolysis? (at the general emergency department, on the CT table, at the stroke unit);(10) What is the size of the nursing team available for thrombolysis? (one nurse, two nurses, more than two nurses); (11) Are there any other factors that could potentially delay thrombolysis? (Please explain).

All 34 questionnaires from the 34 stroke units in Austria were returned and were analyzed in a pseudoanonymized way.

### Statistical Analysis

As a statistical environment R (version 2.11.1) [11] with the package lars [12] was used. The relationship between DNT and several explanatory variables listed in table 1 were modeled by a linear regression. Due to the skewed distribution of the DNT, the log-transformed DNT was used as target variable. The variable subset selection was done using the least absolute shrinkage and selection operator (LASSO) procedure. [13] For each variable subset along the lasso trace the model was refitted without restriction and the Bayesian Information Criterion (BIC) was computed. The final model optimizes the BIC and has been found to be superior to the stepwise forward and the stepwise backward model.

### Standard Protocol Approvals, Registrations, and Patient Consents

The registry is part of a governmental quality assessment program for stroke care in Austria financed by the Federal Ministry of Health. It is based on the federal law promoting quality in health (Gesundheitsqualitätsgesetz). Anonymized data are centrally administrated by the Gesundheit Österreich GmbH and scientific analyses are approved and supervised by an academic review board. Details on this registry have been reported previously [8]–[10].

## Results

Between April 2004 and November 8^th^ 2012, a total of 57991 patients with an acute ischemic stroke were treated in Austrian stroke units. 7498 (13%) of them received a therapy with intravenous thrombolysis. After excluding individuals with a DNT of more than 240 minutes, those with incomplete information about risk factors and modified Rankin scale before admission and individuals with in hospital stroke (ODT ≤0 minutes), 6246 subjects remained and served as the current study population. Baseline characteristics for those with complete dataset are summarized in Table 2. Baseline characteristics for the whole population (n = 7498) do not differ significantly from those with complete dataset (data not shown).

**Table 2:** Description of the study population (N = 6246).

Age, years, median (IQR)	74 (64, 81)
Sex, female, N (%)	3305 (53)
National Institute of Health Stroke Scale (NIHSS), median (IQR)	9 (5, 15)
Onset-to-Door Time (ODT), minutes, median (IQR)	75 (50, 106)
Door-to-needle Time (DNT), minutes, median (IQR)	48 (35, 67)
Onset-to-Needle Time (ONT), minutes, median (IQR)	130 (100, 169)
Hypertension, N (%)	4935 (79)
Diabetes mellitus, N (%)	1282 (21)
Previous stroke, N (%)	1056 (17)
Previous heart attack, N (%)	577 (9)
Hypercholesteremia, N (%)	3247 (52)
Atrial fibrillation, N (%)	2039 (33)
Other cardiac disease, N (%)	1345 (22)
Peripheral artery disease, N (%)	316 (5)
Current smoker, N (%)	1039 (17)
Prestroke disability (mRS 3–5), N (%)	531 (9)
Thrombolysis during working hours, 8–16 h Monday to Friday, N (%)	2587 (41)

IQR = interquartile range; mRS = modified Ranking Scale.

**Figure 1:**
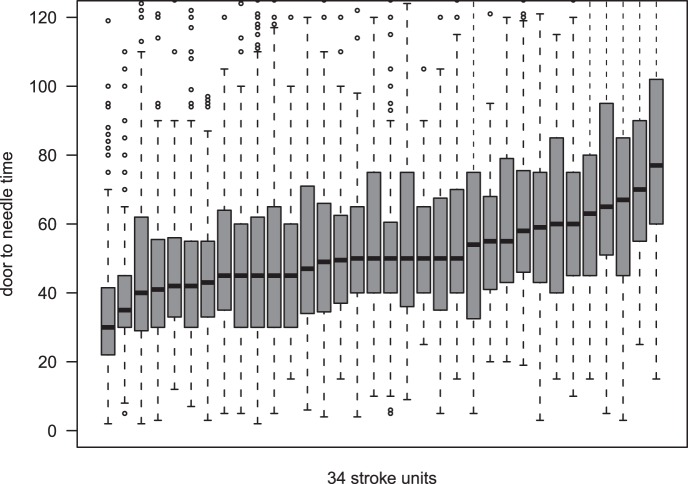
Inter center variability of the door-to-needle times in the 34 stroke units in Austria (box-plots).

The median DNT (2005–2012) in the different centers ranged between 30 and 78 minutes (Figure 1). The proportion of patients receiving rtPA within 60 minutes of stroke onset increased over the years over all stroke centers in Austria from 59% in 2005 to 76% in 2012. In 2012, median DNT ranged between 26 and 71 minutes. Results of the questionnaire of 34 Austrian stroke units are summarized in table 3.

**Table 3:** Results of questionnaire on process measures in Austrian stroke units (n = 34).

What percentage of patients arrive via the pre-notification-system?							
<25%							14.70%
<50%							17.60%
>50%							26.50%
>75%							41.20%
Where are patients admitted to in hospital?							
Directly to the stroke unit						32.40%	
Straight to the emergency department with neurologist on duty						29.40%	
To the general emergency department						38.20%	
Is the stroke unit/emergency department with a neurologist on duty located near the radiology department?							
Yes					82.40%		
No					17.60%		
What is your routine choice of imaging investigation before thrombolysis?							
Plain head CT				55.90%			
Other				44.10%			
Who escorts the patient to the CT/MRI scanner?							
Porters’ services			58.80%				
Doctor on duty			32.40%				
Other			8.80%				
Are there any other investigations performed before thrombolysis? (e.g. ultrasound, …)							
Yes			17.60%				
No			82.40%				
Do you wait for any blood test results to come back for before initiating systemic thrombolysis?							
If the history does not suggest that the patient is on oral anticoagulation thrombolysis is immediately started		23.50%					
Point of care tests are routinely performed		14.70%					
Wait for the full blood count		47.10%					
Other approach		14.70%					
Do you weigh the patient on a scale?							
Yes	14.70%						
No	85.30%						
Where do patients receive thrombolysis?							
General emergency department		11.80%					
CT scanner		2.90%					
Stroke unit		85.30%					
What is the size of the nursing team available for thrombolysis?							
One nurse	32.40%						
Two nurses	58.80%						
More than two nurses	8.80%						

**Figure 2:**
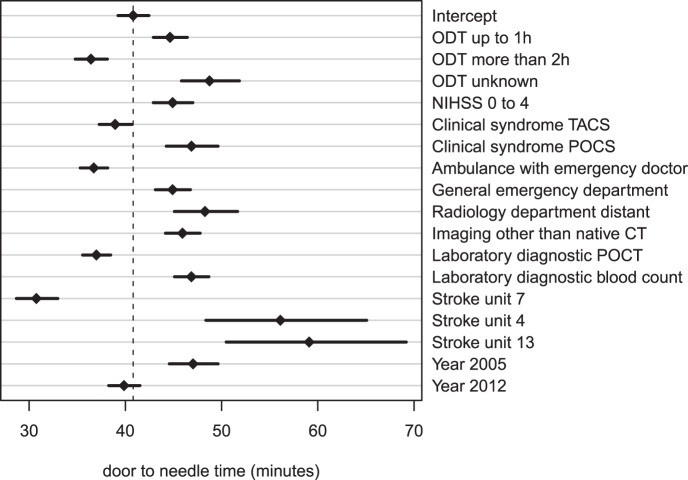
Multiple regression model of case level and center specific factors on the door-to-needle time in Austria. The model contains 18 coefficients (including the intercept) and is based on 5858 observations (adjusted R2 0.14). Since the target variable is log(DNT) the coefficients are not additive in terms of the DNT, but the exp(coefficients) are multiplicative factors in relation to the reference value exp(Intercept).

In a multiple regression model, the impact of defined process measures (according to the structured questionnaire) and patient level data (taken from the Austrian Stroke Unit Registry) on the delay in DNT was quantified. ODT ≤60 minutes, unknown ODT, patients with an NIHSS ≤4 or posterior circulation stroke, initial admission to a general emergency department, a distant radiology department, primary imaging modality other than CT without contrast agent and waiting for the results of the blood count were associated with an increase in DNT whereas an ODT >120 minutes, patients with total anterior circulation *s*troke, recent year of admission, patient transportation with ambulance crew and emergency physician, the use of point of care tests all reduced the DNT (Figure 2). Furthermore three centers yielded a significantly different DNT independent of all the factors mentioned above.

## Discussion

Differences in in-hospital delays for the treatment with thrombolysis in different stroke centers is a well described and important, yet unresolved, issue [6], [7], especially as delays in treatment translate into an adverse outcome [4]. Even though acute stroke care in Austria is highly efficient with shorter OTT and a lower three month mortality than other non-Austrian centers in the SITS registry [14] and with a high proportion of patients treated with rtPA within 60 minutes of arrival to the hospital, we still observe a considerable variability in the DNT between centers.

In accordance with our own and other previous evaluations patient factors like a delay in arrival to the hospital (ODT) and stroke severity influence the DNT. [5]–[8] Interestingly other factors previously reported to cause delays in the DNT like age, sex and the number of patients treated with intravenous thrombolysis annually [6], [7] did not independently alter the in-hospital delays in Austria.

Our analysis shows that the highest in-hospital delays are associated with imaging. Especially a radiology department distant from the location where the stroke patient is treated and any imaging other than plain CT (in accordance with Meretoja et al [5]) prolonged the DNT considerably.

Another substantial prolongation of the DNT was associated with lab analyses in our evaluation. Given the low prevalence of coagulation disorders in stroke patients without a clear medical history [15] thrombolytic therapy should not be delayed while awaiting the lab resuls unless there is either a clinical suspicion of bleeding or thrombocytopenia or the patient has receiced heparin or warfarin or the use of anticoagulants is known. [16] The problem of pretreatment with oral anticoagulation or an unclear medication history can be safely overcome by the use of a point of care device to measure the INR [17].

Interestingly, also in patients with a stroke in the posterior circulation and those with a low stroke severity thrombolysis was delayed. This might be due to the fact that many local thrombolysis protocols cover only hemispheric strokes with an NIHSS of 4 to 25. In addition the NIHSS has been reported to underestimate stroke severity in the posterior circulation [18], [19].

Even though we found no association between the experience of a single stroke center and the DNT, this was true in other evaluations [6]. Still direct presentation of a stroke patient to an experienced team (neurological emergeny department or stroke unit) leads to a significant shorter DNT in Austria. This is especially true for those patients accompanied by an emergency physician. In this context a prenotification system to alarm a stroke team has been shown to be of use [5], [20].

Even after correcting for a wide array of process measurements and patient level data, still two stroke units were significantly slower and one significantly faster than all other Austrian centers. We can only hypothize about the possible background like hard to define human factors, lack of resources and referral biases.

Recent literature has shown that interventions aiming to modify processes in acute stroke treatment can [21] but not necessarily have to be [22] efficient. Therefore changes in process measurements should closely be monitored for their effect on patient care. Upcoming intervention studies [23] might help to optimize process measurements.

To the best of our knowledge this is the first study exploring the influence of a combination of center and case specific variables on the DNT of all designated stroke units of a whole country.

The strengths of the study are as follows: First, Austria and its stroke unit network is especially suited to address the questions as the large sample of 57991 patients treated 34 stroke units reflects the acute stroke care of a whole country. Second, bias like incomplete center participation or reduced access to healthcare [4] do not apply to our evaluation as all centers of a whole country were included, all of them offering stroke care free of charge due to a general health insurance in Austria.

Our study has several limitations: First, administrative and clinical registries are limited in the details of clinical information. Second, as every designated stroke center aims to improve patient care, process measures are constantly adapted in every stroke unit. The information on center specific processes was obtained in winter 2011. Even though we did not observe above the average drop in DNT in the three centers with unexplained deviations in in-hospital delays for iv thrombolysis over the years, the same factors remained in the model when our analysis was restricted to the years 2011 and 2012 and a clustered analysis of different years showed a similar impact of the different factors in delaying treatment, we cannot exclude that this bias might have influenced our results (data not shown). Third, we do not have data on how many of our stroke patients were not treated with intravenous thrombolysis, because they were admitted to other wards than stroke unit and therefore were not entered in the registry.

### Conclusion

The median DNT in Austria has constantly decreased over the years to 48 minutes. The seemingly high inter center variability could largely be explained by differences in process measures and patient factors. Based on our analysis several actions might be useful to reduce the DNT: First of all patients should directly be seen by a neurologist at a specialized stroke unit or neurological emergency department. Optimizing time to CT scan and performing the fastest imaging modality in patients with clear onset of symptoms seems crucial. POC devices (for measurement of INR) or initiating thrombolyis before arrival of the lab results in selected patients can significantly reduce delays created by lab analyses. Furthermore standardized protocols, especially for groups of patients with syndromes or conditions leading to uncertainties and thereby to delays, like for example patients with a stroke in the posterior circulation or a low NIHSS, might be useful. One should keep in mind that stroke evaluation and treatment has top priority independent of the ODT. The latter can substantially reduced by education of dispatchers and emergency system. In conclusion, we highly recommend a continuous quality assessment in the context of acute stroke treatment and a regular lively intra- and intercenter discussion of possibilities to optimize processes and patient care. Further studies might be useful to identify interventions and process modifications most suitable to reduce the DNT and to stimulate a global change in the treatment of acute stroke.

### Austrian Stroke-Unit-Registry Collaborators

Eugen Trinka, MD (Christian-Doppler-Clinic, Salzburg, local site investigator); Johannes Sebastian Mutzenbach, MD (Christian- Doppler-Clinic, Salzburg,local site investigator); Christoph Sulzer, MD (Christian-Doppler-Clinic, Salzburg, local site investigator); Walter Schreiber, MD (Donauspital, Vienna, local site investigator); Sabine Torma, MD (Donauspital, Vienna, local site investigator); Regina Katzenschlager, MD (Donauspital, Vienna, local site investigator); Franz Gruber, MD (General Hospital, Linz, local site investigator); Milan R.Vosko, MD (General Hospital, Linz, local site investigator); Gerhard Ransmayr, MD (General Hospital, Linz, local site investigator), Michael Brainin, MD (Hospital Donauregion, Tulln, study coordinator); Claudia Tatschl, MD (Hospital Donauregion, Tulln, local site investigator); Roul Eckhardt, MD (Hospital Donauregion, Tulln, local site investigator); Birgit Glawar, MD (Hospital Göttlicher Heiland,Vienna, local site investigator); Doris Doppelbauer, MD (Hospital Göttlicher Heiland,Vienna, local site investigator); Wolf Muellbacher, MD (Hospital Göttlicher Heiland,Vienna, local site investigator); Dietlind Resch, MD (Hospital Hietzing, Vienna, local site investigator); Martina Mayr, MD (Hospital Hietzing, Vienna, local site investigator) Robert Paur, MD (Hospital Hietzing, Vienna, local site investigator); Otto Berger, MD (Hospital Kaiser Franz-Josef, Vienna, local site investigator);Vera Nussgruber,MD (Hospital Kaiser Franz-Josef, Vienna, local site investigator);Wolfgang Grisold, MD (Hospital Kaiser Franz-Josef, Vienna, local site investigator); Joerg Weber, MD (Hospital Klagenfurt, local site investigator); Gerhard Noisternig, MD (Hospital Klagenfurt, local site investigator); Heinz Kohlfuerst, MD (Hospital Klagenfurt, local site investigator); Klaus Berek, MD (Hospital Kufstein, local site investigator); Markus Mayr, MD (Hospital Kufstein, local site investigator);Stefan Haaser, MD (Hospital Kufstein, local site investigator); Dieter Zeiner, MD (Hospital Mostviertel, Amstetten, local site investigator); Dietmar Schafelner, MD (Hospital Mostviertel, Amstetten, local site investigator); Berthold Kepplinger, MD (Hospital Mostviertel, Amstetten, local site investigator); Marc Rus, MD (Hospital Oberwart, local site investigator);Barbara Muellauer, MD (Hospital Oberwart, local site investigator); Franz Hoeger, MD (Hospital Oberwart, local site investigator); Christian Eggers, MD (Hospital of the Mercy Friars Linz, local site investigator);Christof Bocksrucker, MD (Hospital of the Mercy Friars Linz, local site investigator); Erich Gatterbauer, MD (Hospital Otto Wagner, Vienna, local site investigator);Andrea Hackenbuchner, MD (Hospital Otto Wagner, Vienna, local site investigator); Christian Prainer, MD (Hospital Rudolfstiftung, Vienna, local site investigator);Ursula Paukner, MD (Hospital Rudolfstiftung, Vienna, local site investigator); Elisabeth Fertl, MD (Hospital Rudolfstiftung, Vienna, local site investigator); Herbert Koller, MD (Hospital Sigmund Freud, Graz, local site investigator); Wolfgang Doppler, MD (Hospital Sigmund Freud, Graz, local site investigator); Julia Ferrari, MD (Hospital St. John of God Vienna, local site investigator);Agathe Flamm-Horak, MD (Hospital St. John of God Vienna, local site investigator);Wilfried Lang, MD (Hospital St. John of God Vienna, study coordinator); Nenad Mitrovic, MD (Hospital Vöcklabruck, local site investigator); Thomas Salletmayr, MD (Hospital Vöcklabruck, local site investigator); Monika Grunenberg, MD (Hospital Vöcklabruck, local site investigator); Franz Aichner, MD (Hospital Wagner-Jauregg, Linz, study coordinator);Hanspeter Haring, MD (Hospital Wagner-Jauregg, Linz, local site investigator); Raffi Topakian, MD (Hospital Wagner-Jauregg, Linz, local site investigator); Christian Bancher, MD (Hospital Waldviertel Horn, local site investigator);Konstantin Prass, MD (Hospital Waldviertel Horn, local site investigator); Andreas Doppelbauer, MD (Hospital Weinviertel Mistelbach, local site investigator); Stefan Pingitzer, MD (Hospital Weinviertel Mistelbach, local site investigator); Manfred Eder; MD (Hospital Weinviertel Mistelbach, local site investigator);Isabelle Csmarich, MD (Hospital Wiener Neustadt, local site investigator);Andrea Hager-Seifert,MD (Hospital Wiener Neustadt, local site investigator); Franz Fazekas, MD (Medical University,Graz, local site investigator); Kurt Niederkorn, MD (Medical University,Graz,study coordinator) Susanna Horner, MD (Medical University,Graz,local site investigator); Martin Furtner, MD (Medical University, Innsbruck, local site investigator);Phillip Werner, MD (Medical University, Innsbruck, local site investigator); Georg Wille, MD (Medical University, Innsbruck, local site investigator); Claude Alf, MD (Neurological Center Rosenhügel, Hospital Hietzing Vienna - 1st Dept. of Neurology, local site investigator);Georg Dimitriadis, MD (Neurological Center Rosenhügel, Hospital Hietzing Vienna - 1st Dept. of Neurology, local site investigator); Manfred Schmidbauer, MD (Neurological Center Rosenhügel, Hospital Hietzing Vienna - 1st Dept. of Neurology, local site investigator); Marion Vigl, MD (Neurological Center Rosenhügel, Hospital Hietzing Vienna - 2nd Dept. of Neurology, local site investigator);Christa Artner, MD (Neurological Center Rosenhügel, Hospital Hietzing Vienna - 2nd Dept. of Neurology, local site investigator); Christoph Baumgartner, MD (Neurological Center Rosenhügel, Hospital Hietzing Vienna - 2nd Dept. of Neurology, local site investigator); Veronika Dorda, MD (Wilhelminen Hospital, Vienna, local site investigator); Gerhard Daniel, MD (Wilhelminen Hospital, Vienna, local site investigator) Peter Thun, MD (Wilhelminen Hospital, Vienna, local site investigator);Josef Grossmann, MD (Hospital Lienz, local site investigator);Gabi Morgenstern, MD (Hospital Lienz, local site investigator); Nadja Wendlinger, MD (Hospital Lienz, local site investigator); Gesundheit Österreich GmbH/BIQG (M. Moritz, A. Gollmer, R. Kern, L. Seyfang), Steering Group at the GÖG/BIQG (Head: W. Lang).
